# Impact on inequities in health indicators: Effect of implementing the integrated management of neonatal and childhood illness programme in Haryana, India

**DOI:** 10.7189/jogh.05.010401

**Published:** 2015-06

**Authors:** Sunita Taneja, Shikhar Bahl, Sarmila Mazumder, Jose Martines, Nita Bhandari, Maharaj Kishan Bhan

**Affiliations:** 1 Centre for Health Research and Development, Society for Applied Studies, New Delhi, India; 2 Centre for Intervention Science in Maternal and Child Health, Centre for International Health, University of Bergen, Norway; 3 Ministry of Science and Technology, Government of India, New Delhi, India

## Abstract

**Background:**

A trial to evaluate the Integrated Management of Neonatal and Childhood Illness (IMNCI) strategy showed that the intervention resulted in lower infant mortality and improved infant care practices. In this paper, we present the results of a secondary analysis to examine the effect of the IMNCI strategy on inequities in health indicators.

**Methods:**

The trial was a cluster–randomized controlled trial in 18 primary health centre areas. For this analysis, the population was divided into subgroups by wealth status (using Principal Component Analysis), religion and caste, education of mother and sex of the infant. Multiple linear regression analysis was used to examine inequity gradients in neonatal and post–neonatal mortality, care practices and care seeking, and the differences in these gradients between intervention and control clusters.

**Findings:**

Inequity in post–neonatal infant mortality by wealth status was lower in the intervention as compared to control clusters (adjusted difference in gradients 2.2 per 1000, 95% confidence interval (CI) 0 to 4.4 per 1000, *P* = 0.053). The intervention had no effect on inequities in neonatal mortality. The intervention resulted in a larger effect on breastfeeding within one hour of birth in poorer families (difference in inequity gradients 3.0%, CI 1.5 to 4.5, *P* < 0.001), in lower caste and minorities families, and in infants of mothers with fewer years of schooling. The intervention also reduced gender inequity in care seeking for severe neonatal illness from an appropriate provider (difference in inequity gradients 9.3%, CI 0.4 to 18.2, *P* = 0.042).

**Conclusions:**

Implementation of IMNCI reduced inequities in post–neonatal mortality, and newborn care practices (particularly starting breastfeeding within an hour of birth) and health care–seeking for severe illness. In spite of the intervention substantial inequities remained in the intervention group and therefore further efforts to ensure that health programs reach the vulnerable population subgroups are required.

**Trial Registration:**

Clinicaltrials.gov NCT00474981; ICMR Clinical Trial Registry CTRI/2009/091/000715

In human rights terms, the word equity represents equality and fairness. It is synonymous with the notion of distributive justice, or fair distribution of good things within a society, whether they may be material possessions, access to health care or simply survival. Health equity has been defined as the absence of systematic disparities in health (or its social determinants) between more and less advantaged groups [1].

Health indicators such as infant mortality have improved in India over time but still continue to be differential across gender, caste, wealth, education and geography [2]. For example, the National Family Health Survey 2005–2006 showed that infant mortality was 70 per 1000 live births for the poorest and 29 per 1000 for the least poor, 42 and 62 per 1000 live births for urban and rural areas respectively, and 70 and 26 per 1000 live births for those with illiterate mothers and mothers with 12 or more years of schooling respectively. In the past few years India’s economic growth has been impressive, but neither the distribution of wealth generated by economic growth nor direct investments in health infrastructure and support systems have been equitably distributed. The result is that poorer families are less likely to access maternal and child health services than wealthier ones. In addition to economic inequity in access to health care, there are social inequities as well. For example, girls, infants from lower caste families and those with illiterate mothers are less likely to receive health care than boys, infants from higher caste families and those with mothers who have completed secondary school.

In 2002, implementation of the Integrated Management of Neonatal and Childhood Illness (IMNCI) strategy was started in India. In addition to treatment of common neonatal and childhood illnesses, IMNCI included home visits to all newborns in the first week of life, and community mobilization activities. We conducted a cluster randomized trial to evaluate IMNCI and found that its implementation resulted in 15% lower infant mortality in the intervention clusters. We also found a substantial improvement in the home based newborn care practices such as initiation of breast feeding within an hour, exclusive breast feeding at four weeks, delayed bathing and appropriate cord care, and in treatment seeking practices in the intervention clusters [3].

Most large studies to evaluate the effect of interventions on newborn and child mortality report only overall results, and not the effect in vulnerable population subgroups. We believe that for an intervention shown to be efficacious in a representative population, several factors require attention when translating research findings to program policy; these include intervention impact on vulnerable groups. We therefore hypothesized that IMNCI implementation would result in a reduction of inequity in neonatal and post–neonatal mortality, health care for illness and in newborn care practices. In this paper we present the results of a secondary analysis to examine the extent to which the IMNCI implementation changed the prevailing health inequities.

## METHODS

### Methods of the main trial

The methods of the cluster–randomized trial evaluating IMNCI have been previously published and are briefly summarized below [3].

### Setting

The trial was conducted in 18 rural areas served by primary health centres in district Faridabad, Haryana, India, with a population of 1.1 million. In this setting, about half of the mothers had never been to school; 95% of the women do not work outside home. 25% of the newborns are low birth weight and 60% of sick children sought care from medically unqualified private practitioners [4,5].

### Randomization

In order to randomize the primary health centre areas into intervention and control groups, a baseline survey was conducted and information was obtained on proportion of home deliveries, mothers who had never been to school, population per cluster, and neonatal and infant mortality. The clusters were divided into three strata with 6 clusters each according to their baseline neonatal mortality rates. Ten stratified randomization schemes were generated by an independent epidemiologist, of which seven schemes had a similar neonatal mortality rate, proportion of home births, proportion of mothers never been to school and population size in the intervention and control groups. One of these seven schemes was selected by a computer generated random number and was used to allocate the clusters into intervention and control groups.

### IMNCI intervention

The intervention was designed following the guidelines defined by the Government of India for IMNCI [6–9]. The study activities in the intervention clusters included:

a) Post–natal home visits during the newborn period: Community health workers in the intervention clusters were trained to conduct home visits; counsel mothers on optimal essential newborn care practices, identify illnesses, treat mild illness and refer newborns with danger signs.

b) Improving health worker skills for case management of neonatal and childhood illness: All staff working in the public health facilities were trained in improving their existing skills for management of sick neonates and children. Training was given using the Government of India IMNCI training module. Formal and informal sector private providers also underwent IMNCI orientation sessions.

c) Strengthening the health system to implement IMNCI: Supervision of community health workers was improved, workers were provided performance–based incentives, uninterrupted supplies of essential medicines were ensured through village level depots. To improve community awareness of the available services three monthly women’s group meetings were conducted in each village.

### Routine care

Routine care includes the activities that were provided by the health care system for newborns and children in both intervention and control areas. This care was provided by two types of community health workers (Anganwadi workers and Accredited Social Health Activists or ASHAs), first level health workers (Auxiliary Nurse Midwives) and primary health care physicians. The activities of each category of workers are briefly described below:

**Anganwadi workers**: Their routine care activities included preschool education, supplementary nutrition and growth monitoring, largely delivered at Anganwadi centres. Their IMNCI–specific activity (only in intervention areas) was to make home visits after birth to promote optimal newborn care practices.

**Accredited Social Health Activists (ASHAs):** Their routine care activities included promotion of antenatal care, hospital births and immunization and contraception services. Their IMNCI–specific activities (only in intervention areas) were to conduct women’s group meetings to promote newborn care and to treat minor illnesses using the IMNCI algorithm.

**Auxiliary Nurse Midwives (ANMs):** Their routine care activities included provision of immunization, family planning, antenatal care, first level treatment of children with illness and conduction of deliveries. Their IMNCI–specific activity (only in intervention areas) was to treat newborn and childhood illnesses using the IMNCI algorithm.

**Primary health care physicians:** Their routine care activities included provision of outpatient treatment of childhood illnesses. Their IMNCI–specific activity (only in intervention areas) was to treat newborn and childhood illnesses using the IMNCI algorithm.

### Outcome measurement

The primary outcomes of the trial were neonatal and infant mortality, and the secondary outcomes included newborn care practices and care–seeking for illness. The intervention was initiated in January 2007, and data collection for outcome measurement was started in January 2008.

The overall sample size of the study was about 30 000 live births per group, which was calculated for ascertaining a 20% difference in neonatal and infant mortality, the primary outcomes of the study. All live births in the intervention and control clusters were visited on day 29 (for ascertaining neonatal mortality) and at 6 and 12 months of age (for ascertaining post–neonatal mortality). Households in the intervention and control areas were allocated to one of the 110 study field workers who were not involved with IMNCI implementation. The workers visited the allocated households every month to identify new pregnancies and inquire about the outcome of previously identified pregnancies. All live births identified by the workers were entered into a database, which was used to generate the due dates to follow up these infants by making home visits. All households with live births were visited on day 29 and at ages 3, 6, 9, and 12 months to document the vital status of the infant by the worker to whom the household was allocated. The worker confirmed the identification of the infant through a set of questions before asking about the health status of the infant. These surveillance workers were not told the intervention status of the clusters. The follow–up procedures were identical in intervention and control clusters. Information was also obtained from all enrolled infants about socio–demographic characteristics, and possession of assets at enrolment.

Secondary outcomes, including newborn care practices and treatment seeking for illness, were ascertained in a subset of enrolled infants at day 29 of life. These outcomes were assessed through an interview by a research assistant with the primary caregiver that lasted 45 minutes to an hour. The sample size for these outcomes was 6200 per group, which was calculated to ascertain at least a 10% absolute difference in care seeking from an appropriate provider for neonatal illness. A random sample of enrolled infants in both the intervention and control clusters was selected for ascertaining secondary outcomes in the following manner. All live births identified by the surveillance workers were entered into a database. Dates for their 29–day visit were generated using a computer program. At the same time, one of five enrolled infants was randomly selected by the computer program for an interview for secondary outcomes. The identification numbers of infants selected for interview were communicated to the research assistants of the secondary outcome assessment team a day before the scheduled interview.

### Ethical considerations

The study was approved by the ethics review committee of the Society for Applied Studies and World Health Organization. Permissions were also obtained from the state and district authorities. Informed consent was taken from the women with a live birth prior to the first interview. Oversight to the study was provided by a study advisory group and Data Safety Monitoring Board (DSMB).

### Secondary analysis for ascertaining impact on equity

Analysis was performed using Stata software version 11 (StataCorp, College Station, TX, USA) and the methods are described below.

### Population subgroups

The infants in intervention and control clusters were divided into subgroups based on their families’ wealth, religion and caste, mother’s years of schooling and the sex of the infant. The wealth of an individual was determined by a wealth index created using primary component analysis based on all of the assets owned by a household. The fact that a household did not own a particular asset that was generally associated with poor households was also used in the calculation of wealth index. The following variables from the initial survey were used to determine the assets owned by a household: the source of drinking water, use of electricity, type of sanitation, type of cooking fuel used, construction materials used for roof, floor and walls of the house, ownership of items like mattress, a pressure cooker, a chair, a cot/bed, a table, an electric fan, a radio/transistor, a black and white television, a colour television, a sewing machine, a mobile telephone, any other telephone, a computer, a refrigerator, a watch or clock, a bicycle, a motorcycle or scooter, an animal–drawn cart, a car, a water pump, a thresher, a tractor, house ownership; number of household members per sleeping room; ownership of a bank or post–office account. An asset score with a mean of 0 and standard deviation of 1 was used in the principal component analysis. Using the score from the wealth index the population was divided into five equal wealth quintiles. Religion and caste was classified into upper caste Hindu, lower caste Hindus (scheduled castes and tribes), and non–Hindu. Maternal education was classified as none, 1–9 years, 10–11 and 12 or more years of schooling.

### Inequities in health outcomes

Neonatal mortality, post neonatal mortality, newborn care practices (eg, exclusive breastfeeding within 1 hour) and careseeking from an appropriate provider for danger signs and pneumonia were displayed for intervention and control areas in subgroups by wealth quintiles, religion and caste, maternal education and sex of the infant. We chose to analyze inequities in neonatal and post–neonatal mortality separately because the overall results of IMNCI trial showed that most of the effect of the intervention on infant mortality was attributable to post–neonatal mortality.

In order to visually assess the degree of income–related inequity in the distribution of health outcomes in intervention and control clusters (neonatal deaths, post neonatal deaths, number of infants who initiated breastfeeding within one hour after birth), we used General Lorenz concentration curves. The concentration curve plots the cumulative percentage of the health outcome (y–axis) against the cumulative percentage of the population ranked by wealth quintile, beginning with the poorest, and ending with the richest (x–axis). The curve is expected to be above the diagonal equity line for a negative outcome like mortality indicating that more deaths occur in the poorer than richer quintiles in the population. Conversely, the curve is expected to be below the equity line for a positive outcome such as utilization of health care indicating that relatively lower number of the poorer quintiles has the outcome.

### Effect of the intervention on inequity

The results were analyzed through a multiple linear regression model with a health outcome (neonatal mortality, post–neonatal mortality, exclusive breastfeeding within 1 hour and care seeking from an appropriate provider for a danger sign) as the dependent variable and population subgroups (by wealth quintile, religion and caste, level of education of the mother and sex of infant) as the independent variable. This multiple regression model was adjusted for cluster design and possible confounders such as distance of the cluster from the highway and percent of home births in the cluster. Additional covariates were the intervention group (intervention or control) and an interaction term of the intervention with the population subgroup (eg, wealth quintile × intervention group). The regression coefficient of this interaction term, which reflects the difference in inequities between the intervention and control groups, was the main indicator of the effect of the intervention on equity.

## RESULTS

### Overall results of the IMNCI trial

The overall results of the trial have been published previously [3] but are briefly described here in order to provide the reader an overview of the overall impact of the intervention before presenting the results related to inequities.

A total of 60 702 infants were enrolled into the trial. There were some differences between the intervention and control clusters at baseline. The control clusters had features of urbanization; a higher proportion of houses had private toilets (46% vs 38%) and a lower proportion possessed ‘below poverty line’ card, the families in the control clusters were nearer to the highway than families in the intervention areas (7.0 km vs 15.3 km) and had lower proportion of home births (65.9% vs 71.9%).

Overall, the infant mortality rate was significantly lower in the intervention clusters than in the control clusters (adjusted hazard ratio 0.85, 95% CI 0.77 to 0.94). The adjusted hazard ratio for neonatal mortality rate was 0.91 (0.80 to 1.03) and that for post–neonatal mortality was 0.76 (0.67 to 0.85). The intervention clusters had significant improvement in newborn and infant care practices. For example, almost 41% of the caregivers in the intervention clusters reported starting breastfeeding within an hour of birth, compared with 11.2% in the control clusters (odds ratio 5.21, 95% CI 4.33 to 6.28).

### Population sub–groups in intervention and control clusters

The proportion of poorer households and mothers with no formal schooling was slightly lower in the intervention compared with control clusters. Sex was equally distributed across intervention and control clusters. The largest difference between study groups was in the proportion of non–Hindus (8.9% in intervention and 24.3% in control clusters, **Table 1**).

**Table 1:** Population sub–groups in intervention and control clusters

Characteristics of families of recruited infants	Intervention clusters (%)	Control clusters (%)
**Wealth quintiles of household:**	n = 29 589	n = 30 604
Poorest	5620 (19.0)	6421 (20.9)
Very poor	5380 (18.2)	6660 (21.8)
Poor	5818 (19.7)	6222 (20.3)
Less poor	6039 (20.4)	6001 (19.6)
Least poor	6732 (22.8)	5300 (17.3)
**Mother’s education level:**	n = 29 545	n = 30 499
None	11 220 (38.0)	12 846 (42.1)
1–9 years of schooling	12 238 (41.4)	11 604 (38.1)
10–11 years of schooling	3460 (11.7)	3405 (11.2)
≥12 years of schooling	2627 (8.9)	2644 (8.7)
**Sex:**	n = 29 667	n = 30 813
Male	15 623 (52.7)	16 252 (52.7)
Female	14 044 (47.3)	14 561 (47.3)
**Religion/caste:**	n = 29 565	n = 30 577
Upper caste	19 407 (65.6)	16 122 (52.7)
Schedule caste/schedule tribe	7532 (25.5)	7013 (22.9)
Non–Hindu	2626 (8.9)	7442 (24.3)

### Inequities in health outcomes in the control population

There were large inequities in health outcomes across different population subgroups. Mortality outcomes were substantially higher among more vulnerable population sub–groups. For instance, in the control clusters, post–neonatal mortality was 41.7 per 1000 in the poorest and 14.0 per 1000 live births for the least poor, 36.5 and 18.5 per 1000 live births in non–Hindus and upper caste Hindus, 32.3 and 20.8 per 1000 live births among female and male infants, 36.3 and 9.8 per 1000 live births in infants of mothers with no formal schooling and those with 12 years or more of schooling. On the other hand, access to health care was lower in the vulnerable population subgroups. In the control clusters, 17.1% and 42.7% of neonates from the poorest and least poor households were taken for health care from an appropriate provider when they had a danger sign. The corresponding values for the same outcome were 12.3% and 38.4% for non–Hindu and upper–caste Hindus, 19.3% and 36.4% of female and male infants, 19.6% and 51.4% of infants of mothers with no formal schooling and 12 or more years of schooling (**Tables 2**** to ****5**).

**Table 2:** Effect of intervention on inequities in neonatal mortality in the intervention and control clusters

Subgroups (total infants in intervention/control clusters)	No. of deaths (NMR/1000)		Difference in inequity gradient (95% CI)*	P–value
	**Intervention (n = 29 667)**	**Control (n = 30 813)**		
**Wealth quintile:**				
Poorest (5620/6421)	293 (52.1)	348 (54.2)		
Very poor (5380/6660)	248 (46.1)	334 (50.2)		
Poor (5818/6222)	252 (43.3)	224 (36.0)		
Less poor (6039/6001)	241 (39.9)	218 (36.3)		
Least poor (6732/5300)	208 (30.9)	177 (33.4)		
Change in NMR/subgroup (inequity gradient)	–3.6 (–6.0 to –1.2)	–4.1 (–5.9 to –2.3)	0.5 (–2.0 to 2.9)	0.681
**Religion and caste:**				
Hindu scheduled caste/tribe (7532/7013)	352 (46.7)	330 (47.1)		
Non–Hindu (2626/7442)	117 (44.6)	322 (43.3)		
Hindu Upper Caste (19 407/16 122)	773 (39.8)	648 (40.2)		
Change in NMR/subgroup (inequity gradient)	–0.2 (–3.6 to 3.3)	0.2 (–3.7 to 4.0)	–0.3 (–4.8 to 4.1)	0.872
**Gender:**				
Female (14 044/14 561)	577 (41.1)	614 (42.2)		
Male (15 623/16 252)	667 (42.7)	712 (43.8)		
Change in NMR/subgroup (inequity gradient)	1.9 (–4.9 to 8.7)	2.0 (–3.1 to 7.2)	–0.1 (–8.7 to 8.4)	0.974
**Mother's years of schooling:**				
None (11 220/12 846)	537 (47.9)	626 (48.7)		
1–9 years (12 238/11 604)	501 (40.9)	478 (41.2)		
10–11 years (3460/3405)	117 (33.8)	127 (37.3)		
12+ years (2627/2644)	83 (31.6)	57 (21.6)		
Change in NMR/subgroup (inequity gradient)	–2.9 (–5.1 to –0.71)	–4.8 (–8.2 to –1.4)	1.9 (–1.9 to 5.7)	0.296

NMR – neonatal mortality, CI – confidence interval

*Multiple linear regressions adjusted for cluster design and potential confounders (distance of nearest point from PHC to highway, percent of home births, and years of schooling of mother, gender, religion and caste and wealth quintile).

**Table 5:** Effect of intervention on inequities in care–seeking from an appropriate provider for a danger sign during the neonatal period in intervention and control clusters

Subgroups (newborns with danger signs in intervention/control groups)	N (%) taken for care to an appropriate provider		Difference in inequity gradients (95% CI)*	P–value
	**Intervention (n = 1010)**	**Control (n = 1269)**		
**Wealth quintile:**				
Poorest (185/257)	60 (32.4)	44 (17.1)		
Very poor (164/258)	58 (35.4)	47 (18.2)		
Poor (187/256)	89 (47.6)	86 (33.6)		
Less poor (208/250)	100 (48.1)	91 (36.4)		
Least poor (264/246)	165 (62.5)	105 (42.7)		
Change in % taken for appropriate care/subgroup (inequity gradient)	4.6 (2.8 to 6.4)	4.0 (2.5 to 5.5)	0.6 (–1.6 to 2.8)	0.554
**Religion and caste:**				
Schedule caste and tribe (254/304)	97 (38.2)	84 (27.6)		
Non–Hindu (79/308)	18 (22.8)	38 (12.3)		
Hindu Upper Caste (677/653)	359 (53.0)	251 (38.4)		
Change in % taken for appropriate care/subgroup (inequity gradient)	3.9 (–0.2 to 7.9)	2.8 (0.1 to 5.4)	1.1 (–3.9 to 6.1)	0.653
**Gender:**				
Female (400/514)	165 (41.3)	99 (19.3)		
Male (610/755)	309 (50.7)	275 (36.4)		
Change in % taken for appropriate care/subgroup (inequity gradient)	8.3 (1.6 to 15.1)	17.6 (11.4 to 23.8)	–9.3 (–18.2 to –0.4)	0.042
**Mother's years of schooling:**				
None (405/555)	156 (38.5)	109 (19.6)		
1–9 years (395/447)	188 (47.6)	144 (32.2)		
10–11 years (119/157)	67 (56.3)	65 (41.4)		
12+ years (91/109)	63 (69.2)	56 (51.4)		
Change in % taken for appropriate care/subgroup (inequity gradient)	5.5 (1.5 to 9.4)	6.5 (2.4 to 10.6)	–1.0 (–6.5 to 4.4)	0.694

CI – confidence interval

*Multiple linear regressions adjusted for cluster design and potential confounders (distance of nearest point from PHC to highway, percent of home births, years of schooling of mother, gender, religion and caste and wealth quintile). Appropriate care provider: Physicians in government and private facilities, auxiliary nurse midwife, Anganwadi worker, or accredited social health activist.

### Effect of the IMNCI intervention on inequities in health indicators

**Figure 1:**
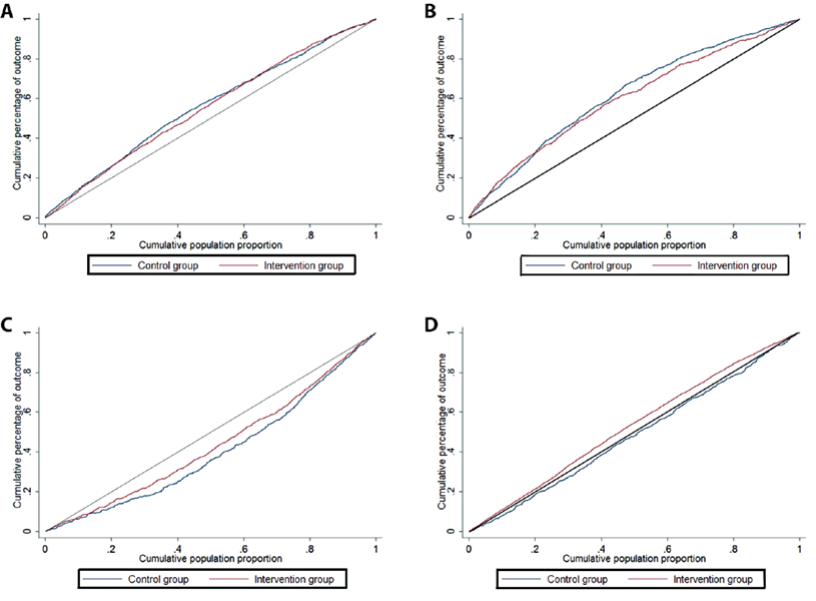
Concentration curves for different health outcomes and wealth quintiles. A. Early initiation of breastfeeding. B. Care seeking for danger signs from an appropriate provider. C. Neonatal mortality D. Post–neonatal mortality.

Inequities in health outcomes in intervention and control clusters are graphically depicted in **Figure 1**. The IMNCI intervention does not appear to substantially change inequities in neonatal mortality but the concentration curves for post–neonatal mortality indicate greater equity in the intervention clusters compared with the control clusters. The intervention clusters also show a more equitable distribution of early initiation of breastfeeding and seeking care for danger signs from an appropriate provider.

The results of multiple linear regression analysis confirmed that IMNCI intervention did not have a significant effect on inequities in neonatal mortality by wealth status, religion and caste, maternal education or gender. The inequities in neonatal mortality were similar in intervention and control groups across different subgroups after adjustment for cluster design and potential confounders (**Table 2**).

The inequities in post–neonatal infant mortality by wealth status were significantly lower in the intervention as compared to control clusters. Post–neonatal mortality was lower by 4.9 per 1000 per wealth quintile when going from the poorest to the least poor in the control group, but only by 2.8 per 1000 per quintile in the intervention group (adjusted difference in gradients 2.2 per 1000, 95% confidence interval 0 to 4.4 per 1000, *P* = 0.053). There were similar differences in gradients across subgroups by religion and caste, gender and years of schooling of the mother but these differences were not statistically significant (**Table 3**).

**Table 3:** Effect of intervention on inequities in post–neonatal mortality in the intervention and control clusters

Subgroups (total infants in intervention/control cluster)	No. of deaths (rate/1000)		Difference in inequity gradients (95% CI)*	P–value
	**Intervention (n = 29 667)**	**Control (n = 30 813)**		
**Wealth quintile:**				
Poorest (5620/6421)	214 (38.1)	268 (41.7)		
Very poor (5380/6660)	134 (24.9)	219 (32.9)		
Poor (5818/6222)	119 (20.5)	153 (24.6)		
Less poor (6039/6001)	111 (18.4)	91 (15.2)		
Least poor (6732/5300)	100 (14.9)	74 (14.0)		
Change in mortality rate/subgroup (inequity gradient)	–2.8 (–4.2 to –1.3)	–4.9 (–7.0 to –2.8)	2.2 (0 to 4.4)	0.053
**Religion and caste:**				
Schedule caste and tribe (7532/7013)	229 (30.4)	233 (33.2)		
Non–Hindu (2626/7442)	69 (26.3)	272 (36.5)		
Hindu Upper Caste (19 407/16 122)	379 (19.5)	298 (18.5)		
Change in mortality rate/subgroup (inequity gradient)	–1.8 (–4.1 to 0.51)	–4.8 (–7.7 to –1.8)	3.0 (–0.6 to 6.6)	0.101
**Gender:**				
Female (14 044/14 561)	392 (27.9)	471 (32.3)		
Male (15 623/16 252)	289 (18.5)	338 (20.8)		
Change in mortality rate/subgroup (inequity gradient)	–9.1 (–12.2 to –6.0)	–10.8 (–14.7 to –6.9)	1.7 (–3.2 to 6.6)	0.479
**Mother's years of schooling:**				
None (11 220/12 846)	355 (31.6)	466 (36.3)		
1–9 years (12 238/11 604)	247 (20.2)	261 (22.5)		
10–11 years (3460/3405)	52 (15.0)	45 (13.2)		
12+ years (2627/2644)	24 (9.1)	26 (9.8)		
Change in mortality rate/subgroup (inequity gradient)	–4.0 (–6.4 to –1.5)	–5.9 (–8.1 to –3.7)	2.0 (–1.3 to 5.2)	0.222

CI – confidence interval

*Multiple linear regressions adjusted for cluster design and potential confounders (distance of nearest point from PHC to highway, percent of home births, years of schooling of mother, gender, religion and caste and wealth quintile).

Among all the outcomes examined in this analysis, inequities in the control group were the smallest for the practice of initiating breastfeeding within 1 hour of birth. The IMNCI intervention substantially increased the prevalence of this practice, and had greater benefit for the more vulnerable population subgroups resulting in inequity gradients that favored infants from poorer families (difference in gradients between intervention and control clusters 3.0%, CI 1.5 to 4.5, *P* < 0.001), lower caste Hindus and non–Hindus (difference in gradients 3.9%, CI 1.8 to 6.0, *P* < 0.001) and mothers with fewer years of schooling (difference in gradients 5.4%, CI 3.4 to 7.4, *P* < 0.001). This pattern of beneficial effects was not seen by infant sex, with boys and girls benefitting equally by the intervention (**Table 4**).

**Table 4:** Effect of intervention on inequities in breastfeeding initiation within 1 h of birth (as reported by the mother) in intervention and control clusters

Subgroups (total infants in intervention/control clusters)	No. breastfed in first hour (%)		Difference in inequity gradients (95% CI)*	P–value
	**Intervention (n = 6204)**	**Control (n = 6163)**		
**Wealth quintile:**				
Poorest (1201/1231)	527 (43.9)	127 (10.3)		
Very poor (1089/1299)	510 (46.8)	154 (11.9)		
Poor (1182/1278)	517 (43.7)	139 (10.9)		
Less poor (1276/1222)	497 (38.9)	140 (11.5)		
Least poor (1452/1122)	475 (32.7)	128 (11.4)		
Change in % initiated breastfeeding early/subgroup (inequity gradient)	–2.8 (–4.2 to –1.1)	0.4 (–0.3 to 1.0)	–3.0 (–4.5 to –1.5)	<0.001
**Religion and caste:**				
Schedule caste and tribe (1556/1469)	718 (46.1)	193 (13.1)		
Non–Hindu (526/1420)	238 (45.3)	93 (6.6)		
Hindu Upper Caste (4119/3254)	1569 (38.1)	399 (12.3)		
Change in % initiated breastfeeding early/subgroup (inequity gradient)	–3.4 (–5.2 to –1.7)	–0.5 (–1.2 to 2.1)	–3.9 (–6.0 to –1.8)	<0.001
**Gender:**				
Female (2893/2845)	1168 (40.4)	323 (11.4)		
Male (3310/3318)	1358 (41.0)	366 (11.0)		
Change in % initiated breastfeeding early/subgroup (inequity gradient)	–0.8 (–2.0 to 3.6)	–0.2 (–2.3 to 1.9)	–1.0 (–2.5 to 4.5)	0.542
**Mother's years of schooling:**				
None (2465/2687)	1068 (43.3)	237 (8.8)		
1–9 years (2548/2260)	1061 (41.6)	301 (13.3)		
10–11 years (642/637)	253 (39.4)	95 (14.9)		
12+ years (547/574)	144 (26.3)	56 (9.8)		
Change in % initiated breastfeeding early/subgroup (inequity gradient)	–3.1 (–4.9 to –1.3)	2.2 (0.8 to 3.7)	–5.4 (–7.4 to –3.4)	<0.001

CI – confidence interval

*Multiple linear regressions adjusted for cluster design and potential confounders (distance of nearest point from PHC to highway, percent of home births, years of schooling of mother, gender, religion and caste and wealth quintile).

Neonates who were taken for health care when they had a danger sign was inequitably distributed in both control and intervention groups. While the IMNCI intervention improved this outcome overall, the differences in inequity gradients in intervention and control clusters were not statistically significant in subgroups by wealth, religion and caste and maternal education. However, the intervention had an impact on reducing inequity in this outcome by infant’s sex. In the control group, only 19.3% of girls compared to 36.3% of severely ill boys were taken for care to an appropriate provider but this difference was reduced in the intervention group with 41.3% of girls and 50.7% of boys taken for appropriate care (difference in gradients 9.3%, CI 0.4 to 18.2, *P* = 0.042).

## DISCUSSION

### Main findings

The beneficial effects of the IMNCI intervention on newborn and infant care practices and survival were equitably distributed among population subgroups. The intervention reduced inequities in post–neonatal mortality between wealth quintiles but did not reduce inequities in neonatal mortality. There was a greater increase in the proportion of neonates who initiated breastfeeding within one hour of birth in the intervention clusters among poorer families, lower caste and minority families and infants of mothers with fewer years of schooling. Care seeking for severe neonatal illness from an appropriate provider improved more for girls reducing gender inequity but inequities in this outcome by wealth, religion and caste and maternal education did not change.

### Potential mechanisms that could explain the results

While there was no attempt to specifically target the poorer and other vulnerable populations in the IMNCI strategy, substantial efforts were made to deliver the intervention to the entire population. We believe that this led to the intervention being delivered to a large proportion of vulnerable population subgroups. These vulnerable population subgroups were also more likely to respond positively to counselling advice as evidenced by a greater improvement of appropriate practices like early initiation of breastfeeding among them because it is least demanding in terms of resources on the mother/family. Availability of appropriate health care close to home resulted in improved care seeking for girls perhaps, due to reduced need of financial resources. It has previously been shown in this population that care for girls is not obtained from hospitals and other health facilities because of lower value placed on girls than that on boys and reluctance of families to use meagre financial resources on the health of girls [10].

Impact on the intervention in reducing inequities in post neonatal mortality is evident but was not observed in neonatal mortality. This lack of impact on inequities in neonatal mortality could be because a high proportion of neonatal deaths occur in the first days of life and are related to maternal health care, which was not part of the IMNCI programme. Further, clinical problems in the neonatal period may develop and evolve rapidly to become serious, and require inpatient care, which was also not included in the IMNCI strategy.

There is no statistically significant effect on differences in post–neonatal mortality between boys and girls. However, the mortality rate in boys was lower in intervention group compared to the control group by 2.3 per 1000, whereas the corresponding difference for girls was 4.4 per 1000. This means that there might be some effect of the improved care seeking in girls on their mortality, but there might other inequities that girls face that limit the effect on the difference in mortality between boys and girls.

### Comparison with other studies that have reported impact of interventions on inequities in neonatal and post neonatal mortality

We could only find one study that reported on the impact of IMCI on inequalities in child health [11]. The effect was mixed. Equity differentials for six child health indicators (underweight, stunting, measles immunization, access to treated and untreated bednets, treatment of fever with antimalarials) improved significantly in IMCI districts compared with comparison districts (*P* < 0.05), while four indicators (wasting, DPT coverage, caretakers’ knowledge of danger signs and appropriate care seeking) improved significantly in comparison districts compared with IMCI districts (*P* < 0.05).

A systematic review published in 2014 summarized evidence about the differential effects of interventions on different socio–demographic groups in order to identify interventions that were effective in reducing maternal or child health inequalities [12]. Eleven of 22 studies included in the review reported on the infant and under–five mortality rate. These studies covered five kinds of interventions: immunization campaigns, nutrition supplement programs, health care provision improvement interventions, demand side interventions, and mixed interventions. The review concluded that the studies on effectiveness of interventions on equity in maternal or child health are limited. The limited evidence showed that the interventions that were effective in reducing inequity included the improvement of health care delivery by outreach methods, using human resources in local areas or provided at the community level nearest to residents and the provision of financial or knowledge support to improve demand side determinants [12]. May be vulnerable groups would benefit more if IMNCI incorporated community based treatment for the less severely ill neonates and leaving referral to health facilities for the severely ill. For neonatal mortality, one of the studies included in the above review reported that participatory women group intervention can substantial reduce socio–economic inequalities in neonatal mortality [13].

### Strengths and weaknesses of this analysis

The IMNCI evaluation study was a cluster randomized effectiveness trial with a large sample size involving about 60 000 births and it was therefore possible to study the effect of the intervention on inequities with reasonable precision. Detailed baseline information was available for all births in intervention and control clusters allowing accurate classification into population subgroups by wealth, religion and caste, sex and level of maternal education. There was an independent and similar measurement of outcomes in intervention and control clusters with very low rates of follow up.

There are a couple of weaknesses of this analysis that merit consideration. There are inherent weaknesses of a subgroup analysis, but examination of equity is only possible with such an analysis. There were some baseline differences between intervention and control clusters which could have resulted in some differences in inequity gradients between them. However, we adjusted the analysis for the baseline characteristics that showed important differences between intervention and control clusters. Finally, it is difficult to separate the effects of different components of the IMCI package, or the effect of “IMNCI home visits” that were made to promote newborn care practices from home visits without any health intervention. However, making home visits with no health intervention in the control group was not possible in this pragmatic cluster randomized trial.

### Conclusions and implications of this paper

The IMNCI strategy, as implemented in the trial, promotes equity in post–neonatal mortality, newborn care practices, particularly for early initiation of breastfeeding and health care seeking for severe illness for some of the vulnerable population subgroups. However, substantial inequities continue to exist despite the intervention and therefore additional efforts are required for health programs like IMNCI not only to reach vulnerable populations such as mothers and children of families with lower socio–economic status, but also to identify and implement interventions that have a greater effect on reducing inequities.
